# Cryo‐EM structure of G‐protein‐coupled receptor GPR17 in complex with inhibitory G protein

**DOI:** 10.1002/mco2.159

**Published:** 2022-09-10

**Authors:** Fang Ye, Thian‐Sze Wong, Geng Chen, Zhiyi Zhang, Binghao Zhang, Shiyi Gan, Wei Gao, Jiancheng Li, Zhangsong Wu, Xin Pan, Yang Du

**Affiliations:** ^1^ Kobilka Institute of Innovative Drug Discovery Shenzhen Key Laboratory of Steroid Drug Discovery and Development School of Medicine The Chinese University of Hong Kong Shenzhen Guangdong China; ^2^ The Chinese University of Hong Kong Shenzhen Futian Biomedical Innovation R&D Center Shenzhen Guangdong China; ^3^ Instrumental Analysis Center Shenzhen University Shenzhen Guangdong China

**Keywords:** cryo‐EM, ECL2, GPCR, GPR17, protein structure

## Abstract

GPR17 is a class A orphan G protein‐coupled receptor (GPCR) expressed in neurons and oligodendrocyte progenitors of the central nervous system (CNS). The signalling of GPR17 occurs through the heterotrimeric Gi, but its activation mechanism is unclear. Here, we employed cryo‐electron microscopy (cryo‐EM) technology to elucidate the structure of activated GPR17‐Gi complex. The 3.02 Å resolution structure, together with mutagenesis studies, revealed that the extracellular loop2 of GPR17 occupied the orthosteric binding pocket to promote its self‐activation. The active GPR17 carried several typical microswitches like other class A GPCRs. Moreover, the Gi interacted with the key residues of transmembrane helix 3 (TM3), the amphipathic helix 8 (Helix8), and intracellular loops 3 (ICL3) in GPR17 to engage in the receptor core. In summary, our results highlight the activation mechanism of GPR17 from the structural basis. Elucidating the structural and activation mechanism of GPR17 may facilitate the pharmacological intervention for acute/chronic CNS injury.

## Graphical abstract

GPR17 is a class A orphan G protein‐coupled receptor. The 3.02 Å resolution structure elucidates the activated GPR17 in complex with the Gαi, Gβ, Gγ, and scFv16 in the ligand‐free state. The ECL2 of GPR17 occupies the orthosteric binding pocket to promote its self‐activation. Meanwhile, the residues D350 and E318 of Gαi form two salt bridges with K331^8.49^ and R252 of GPR17, respectively, which play a prominent role in the assembly of complex.

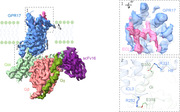

## 1 INTRODUCTION

GPR17 is a class A orphan G protein‐coupled receptor (GPCR), which is phylogenetically related to the families of purine P2Y receptors and cysteinyl leukotriene (CysLT) receptors.^1^ In humans, the GPR17 gene, located on the chromosome 2q21, contains three exons and two open reading frames (ORFs), resulting in two isoforms: a short one with 339 amino acid residues, and a long one with 367 amino acid residues. The isoforms have different functions in the brain tissues.^2^, ^3^

Recently, intensive researches have shown that GPR17 is prominently expressed in central nervous system (CNS), particularly in the oligodendrocyte precursor cells (OPCs).^2^, ^4^, ^5^ Specifically, under normal physiological conditions, GPR17 acts as a regulator of oligodendrocyte differentiation and maturation. As for the expression level of GPR17, the overall trend is to maintain a low level initially and then increase with the transition of OPCs to mature oligodendrocytes and finally decrease again after full maturation.^2^ In contrast, under pathological conditions, GPR17 could be used as a specific marker associated with CNS injury. In the early phase of CNS damage, increased level of GPR17 accelerates the neuronal death. After axonal injury, the GPR17, on the surface of infiltrating macrophages, will gather at the place of nerve tissue so as to aggravate the damage of brain. The GPR17 level is positively correlated with the distance and extent of injury, as well as negatively correlated with the time after injury.^6^ Collectively, GPR17 plays an important role in the identification of CNS injury and the determination of therapeutic approach. In addition, GPR17 is highly expressed in organs susceptible to ischemic damage (e.g., heart, kidney, and brain), which has attracted high attention from researchers. Based on the ischemic stroke models in rats and mice, it has been confirmed that GPR17 can inhibit the progression of ischemic injury, thus considered to be a potential therapeutic target for stroke.^7^, ^8^ Furthermore, GPR17 might also be involved in the regulation of whole‐body energy homeostasis. The mice with systemic or specific knockout of GPR17 display significantly less weight gain during a high‐fat diet. Further analysis suggests that it is mainly attributed to the GPR17/cyclic AMP (cAMP)/lactate signaling axis that regulates the activities of hypothalamic neurons to maintain energy homeostasis.^9^, ^10^ Given these important roles, GPR17 has become a promising target for pharmacological intervention in the neurodegenerative diseases, ischemic damage, and energy metabolism dysregulation.

In 2006, the uracil nucleotides (UDP, UDP‐glucose, and UDP‐galactose) and CysLTs (LTD4 and LTC4) were first identified as the endogenous ligands of GPR17.^11^ The precise activation of GPR17 leads to both adenylyl cyclase inhibition and intracellular calcium increase. However, nucleotides and CysLTs are reported ligands for P2Y and CysLT receptors, and their roles for activating GPR17 were challenged.^12^ In order to further investigate these findings, it is essential to elucidate the underlying activation mechanism of GPR17 by structural biology study. Our research will provide the detailed 3D Structure of GPR17 and facilitate the deorphanization of GPR17.

Previous studies showed that some orphan class A GPCRs are self‐activation and exhibit high level of basal activity without any agonist.^13^, ^14^ They can mediate the cellular signaling immediately after expressing on the cell surface. So far, GPR52 and BILF1 are reported to be activated by their own structural elements.^15^ In this study, we employed cryo‐electron microscopy (cryo‐EM) to resolve the structure of active GPR17‐Gi complex in the absence of ligand and unexpectedly found that the GPR17 is a self‐activated receptor as well. The extracellular loop2 (ECL2) of GPR17 formed a unique loop structure covering the pocket entrance for the cognate ligand. Structural superimposition revealed a high conformational similarity between the active GPR17 and other Gi‐coupled GPCRs except the ECL2 part. Together, the results highlight the self‐activation mechanism of GPR17 from the structural basis, which is mediated by the ECL2.

## 2 RESULTS

### 2.1 Overall structure of the active‐state GPR17

**FIGURE 1:**
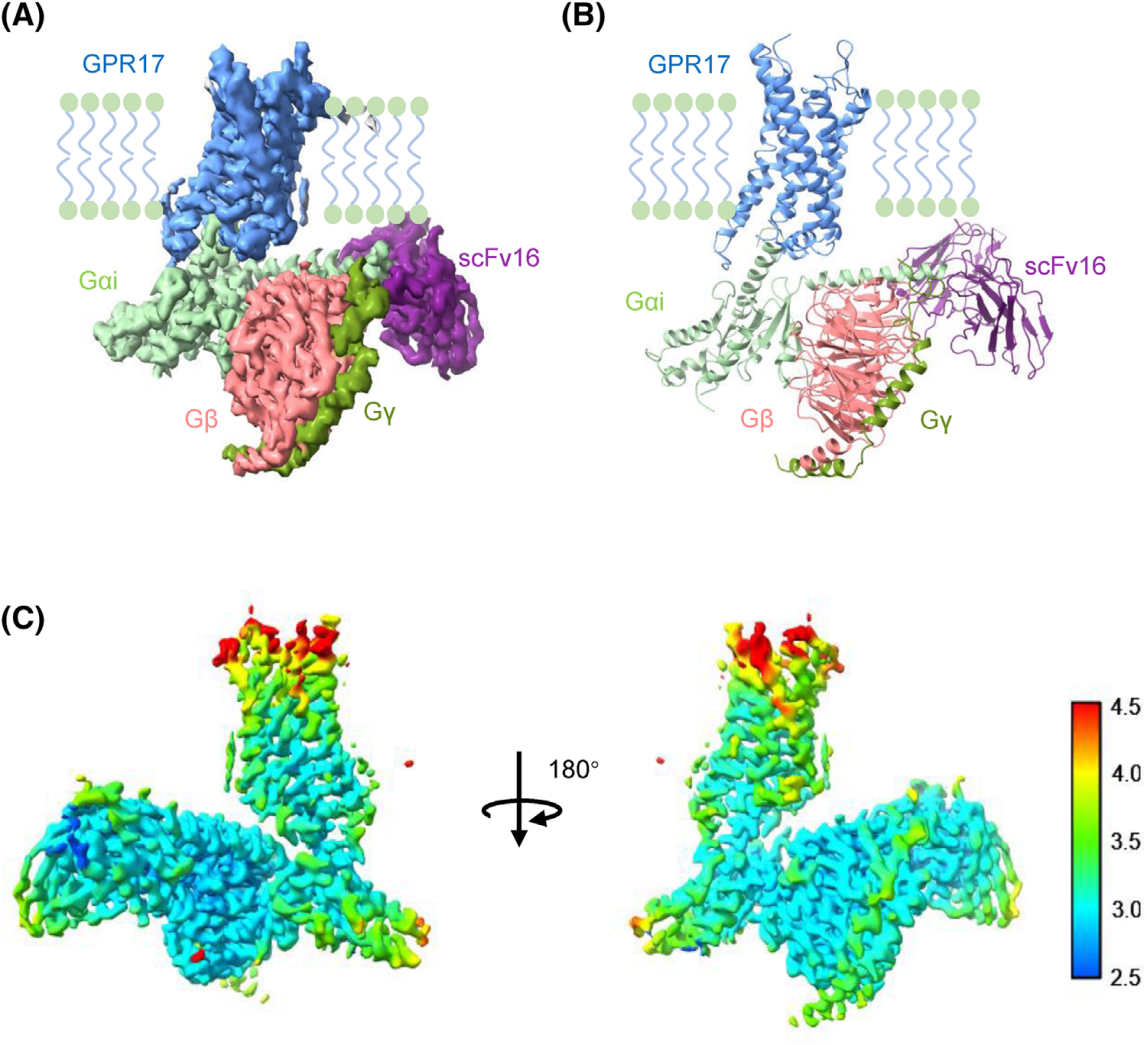
Cryo‐electron microscopy (EM) structure of the GPR17‐Gi complex. (A) Cryo‐EM density map of GPR17‐Gi complex. GPR17, Gαi, Gβ, Gγ, and scFv16 are shown in cornflower blue, pale green, hot pink, green, and purple, respectively. (B) Structural model determined after refinement in the cryo‐EM map. (C) Heatmap showing the spatial resolution of GPR17‐Gi density map

We first solved cryo‐EM structure of human GPR17‐Gi complex in the ligand‐free state. To facilitate complex formation, GPR17 receptor and Gi protein were co‐expressed in Sf9 insect cells. The GPR17‐Gi complex was then purified to homogeneity for cryo‐EM analysis with Titan Krios microscope. The structure revealed the extracellular domains, transmembrane regions, and extracellular domains in complex with Gαi, Gβ, Gγ, and scFv16 at a resolution of 3.02 Å (Figure 1, Figures S1 and S2, and Table S1). The map showed a clear density of the backbone transmembrane helices (TMs), intracellular loops (ICLs), ECLs, and the amphipathic helix 8 (H8). Moreover, the interface residues between GPR17 and Gi (α5‐helix) were clearly defined. Two GPR17 isoforms (long and short) are found in human brain tissue. The short form is 28 amino acids shorter in the N‐terminus of GPR17. We used the long isoform of GPR17 for the complex constitution. However, in the GPR17 map, the whole N‐terminal residues (1‐54) were not resolved, indicating a highly flexible N‐term extracellular domain.

### 2.2 ECL2 occupies the orthosteric pocket

**FIGURE 2:**
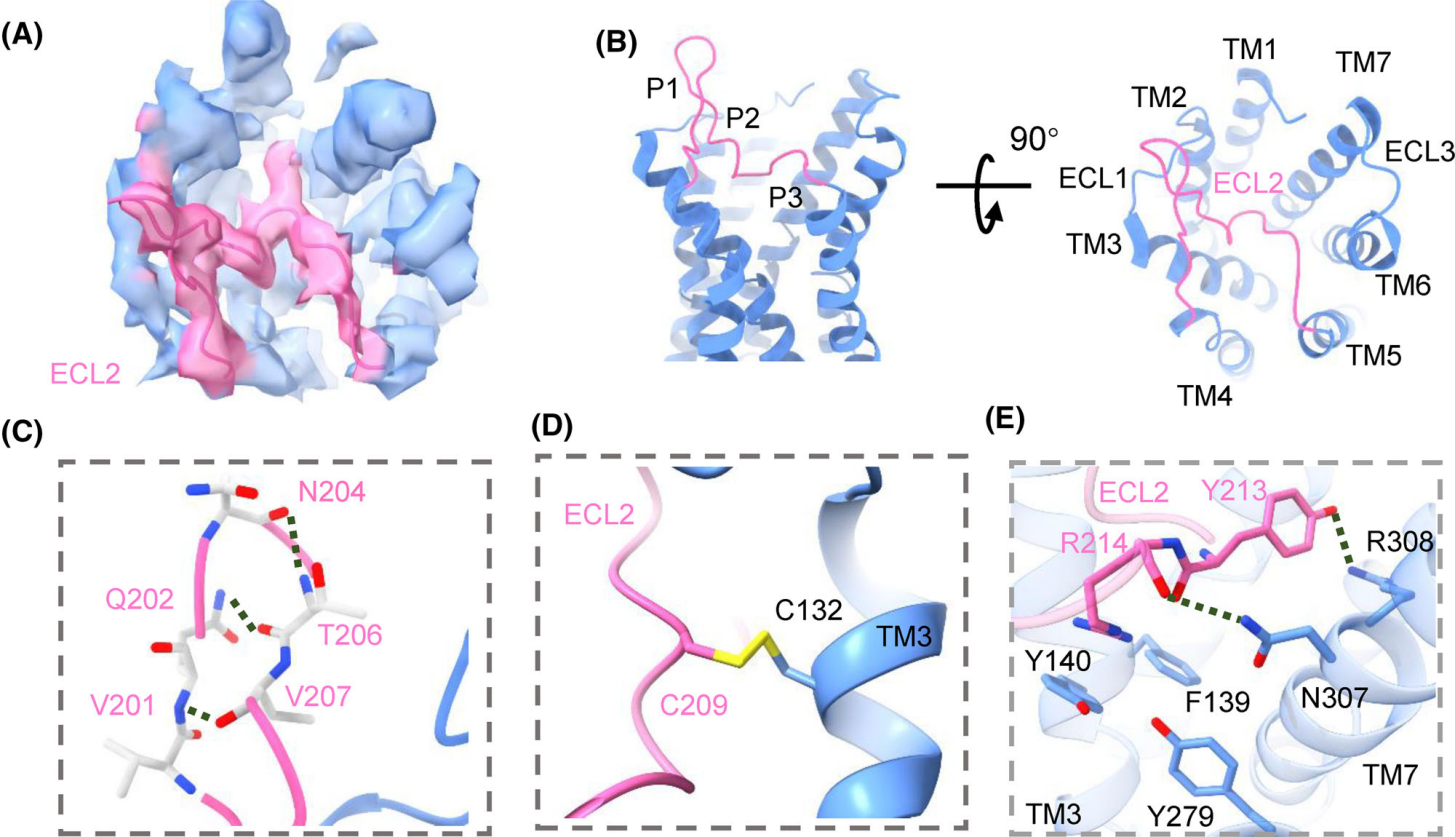
Analysis the ECL2 of GPR17. (A) A top view of the GPR17 map. The ECL2 (hot pink) inserted on the transparent surface presentation of GPR17 map (cornflower blue). (B) Cartoon view of GPR17 (cornflower blue), showing the ECL2 (hot pink) structure overlaid the ligand binding pocket. P1, P2, and P3 segment of ECL2 indicated three parts composed of P198‐T203, N204‐L212, and Y213‐S218, respectively. (C) Magnified view of the ECL2 in the orthosteric binding pocket of GPR17. Key interacting residues were shown as sticks in hot pink, and the hydrogen bonds were shown as dashed lines. (D) The disulfide bond (yellow) between C209 of ECL2 (hot pink) and C132^3.25^ of TM3 (cornflower blue). (E) The interacting residues of ECL2 (hot pink) and TMs (cornflower blue). The hydrogen bonds were shown as dashed lines

**FIGURE 3:**
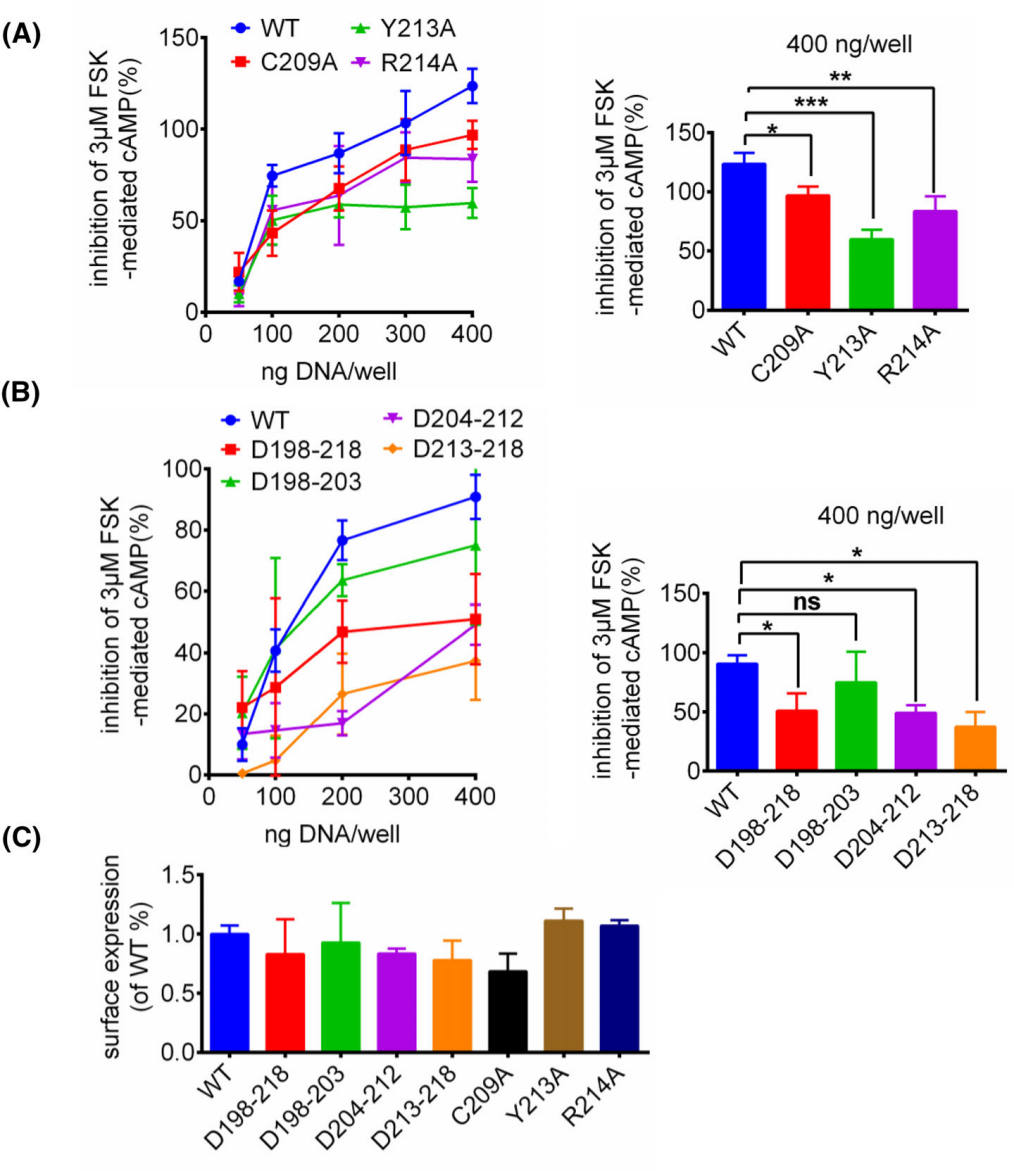
cAMP level and surface expression of WT GPR17 and mutants. (A) cAMP assay (expressed as a percentage of forskolin‐mediated cAMP) identified key residues involved in GPR17. The data were presented as means ± SEM, one‐way analysis of variance (ANOVA), *p < 0.05, **p < 0.01, ***p < 0.001. (B) The effects on cAMP level were compared between WT and ECL2 mutants of GPR17 (D198‐218, D198‐203, D204‐212, D213‐218, were replaced with a six‐residue linker, GGSGGS, respectively). The data represented means ± SEM, one‐way ANOVA, *p < 0.05. (C) Cell surface expression was determined by FACS analysis. WT: wild‐type GPR17. The expression of ECL2 mutants compared to that of GPR17 WT was not significant using one‐way ANOVA. The experiments were performed in triplicates

GPR17 has a unique ECL2 structure, which is not common in other class A GPCR. GPR17 ECL2 is the longest ECL compared to ECL1 (7 residues) and ECL3 (8 residues). The ECL2 covers around 40% of the pocket entrance, which may hinder the entry or egress of ligands (Figure 2A,B). The overall GPR17 ECL2 conformation is a hairpin like structure connected to a loop that inserts into the canonical ligand binding pocket, which is quite similar to the predicted structure in AlphaFold database. ECL2 contains 21 residues (PQTVQTNHTVVCLQLYREKAS), and we divide it into three segments, which are named as P1 segment (P198‐T203), P2 segment (N204‐L212), and P3 segment (Y213‐S218) for further analysis. The whole ECL2 adopts an upward orientation pointing to the extracellular side and inclines toward ECL3. The hairpin like structure (P1 and P2 segment) is stabilized by forming hydrogen bonds with both V201 and V207, Q202 and T206, N204 and T206 between their side chains and backbone carbonyl groups (Figure 2C). The central loop (P3 segment) is connected to TM5, which is immersed in the orthosteric pocket of GPR17. In addition, the conserved disulfide bond between C209 (ECL2) and C132^3.25^ (TM3) provides additional support to stabilize ECL2 in the position (Figure 2D). The residues Y213 and R214 of the P3 segment interact with TM7 residues R308^7.36^ and N307^7.35^ to form hydrogen bonds, which help to lock the receptor in the active conformation (Figure 2E). As shown in the cAMP assays, mutations of C209, Y213, and R214 of ECL2 greatly reduced the constitutive Gi‐mediated cAMP signaling (Figure 3A), which indicates the hydrogen bond formed between ECL2 and TM7 is essential for GPR17‐basal activity. We also replaced ECL2 by six amino acid GS linker (GGSGGS) at different positions, and then the influence on downstream signaling was evaluated (Figure 3B). The mutations of GPR17 ECL2 did not affect the GPR17 expression significantly (Figure 3C),  but reduced the Gi‐mediated receptor signaling profoundly, further supporting the proposal that ECL2 has a key role for GPR17 self‐activation.

### 2.3 Microswitches of GPR17

**FIGURE 4:**
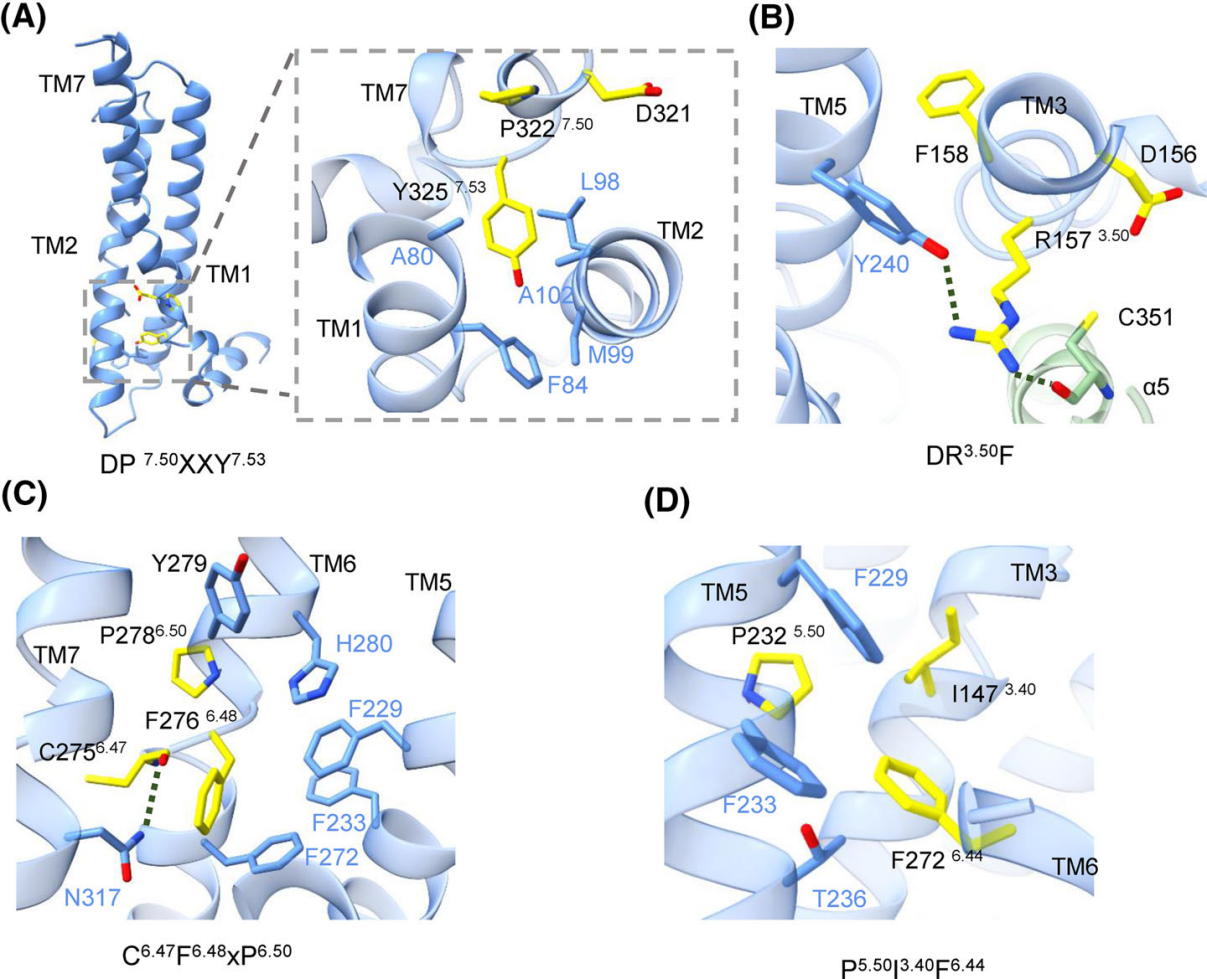
The conformational switches of GPR17. (A) Magnified view of DP^7.50^xxY^7.53^ motif in GPR17. The residues of the motif were shown as yellow sticks, and those interacting with them are shown as cornflower blue sticks. (B) The interacting residues of DR^3.50^F (yellow) with TM5 (cornflower blue) and α5 (pale green) within 4 Å distance. The hydrogen bonds were shown as dashed lines. (C) The interacting residues of C^6.47^F^6.48^xP^6.50^ (yellow) and TM7, TM6, and TM5 (cornflower blue) within 4 Å distance. (D) The interacting residues of P^5.50^I^3.40^F^6.44^ (yellow) and TM5 (cornflower blue) within 4 Å distance

The GPR17 structure showed TM rearrangement, which is consistent with other class A Gi‐engaged GPCRs in an active state. In GPR17, the conserved N^7.49^ residue in the NPxxY motif is replaced by a negatively charged aspartic acid (D^7.49^P^7.50^IMY^7.53^). Y325^7.53^ on TM7 interacts with A80^1.53^, F84^1.57^, L98^2.43^, and M99^2.44^ to form a hydrophobic cluster, contributing to the activation of receptor (Figure 4A). Moreover, the conserved Y^3.51^ in the aspartate‐arginine‐tyrosine (DRY) motif is substituted by F158^3.51^ in GPR17, forming the DRF motif. For inactive class A GPCRs, DRY can form an ionic lock through the oppositely charged residues. The electrostatic lock opens upon ligand‐dependent receptor activation. In GPR17, the ionic lock between D156^3.49^ and R157^3.50^ is opened. R157^3.50^ on TM3 rotates and interacts with Y240^5.58^ of receptor and C351 of Gαi to maintain TM5 and Gi in the position via hydrogen bonds (Figure 4B). In GPR17, the conserved W^6.48^ in the toggle switch is replaced by F^6.48^, forming the C^6.47^F^6.48^xP^6.50^ motif. C275^6.47^, works as a rotameric switch and interacts with N317^7.45^ side chain via hydrogen bond. F276^6.48^ also forms hydrophobic cluster with residues F229^5.47^ and F233^5.51^ of TM5 and F272^6.44^, Y279^6.51^, and H280^6.52^ of TM6. In this scenario, C275^6.47^ and F276^6.48^ may help maintain the hinge structure by interacting with TM5 and TM7 via hydrophobic interactions and hydrogen bonds (Figure 4C). At last, the P^5.50^I^3.40^F^6.44^(PIF) motif interacts with TM3 (I147^3.40^), TM5 (F229^5.47^, P232^5.50^, F233^5.51^), and TM6 (F272^6.44^) via hydrophobic interactions (Figure 4D). Together, the conformation features reveal that GPR17 shares similar microswitches with other class A GPCRs.^16^ GPR17 utilizes the universal macroswitch of class A GPCR on TM6 to open the intracellular cavity through rotation and outward movement of the cytoplasmic half of TM6 to accommodate Gi‐coupled.

### 2.4 Comparison of ECL2 between GPR17 with other self‐activated GPCRs

**FIGURE 5:**
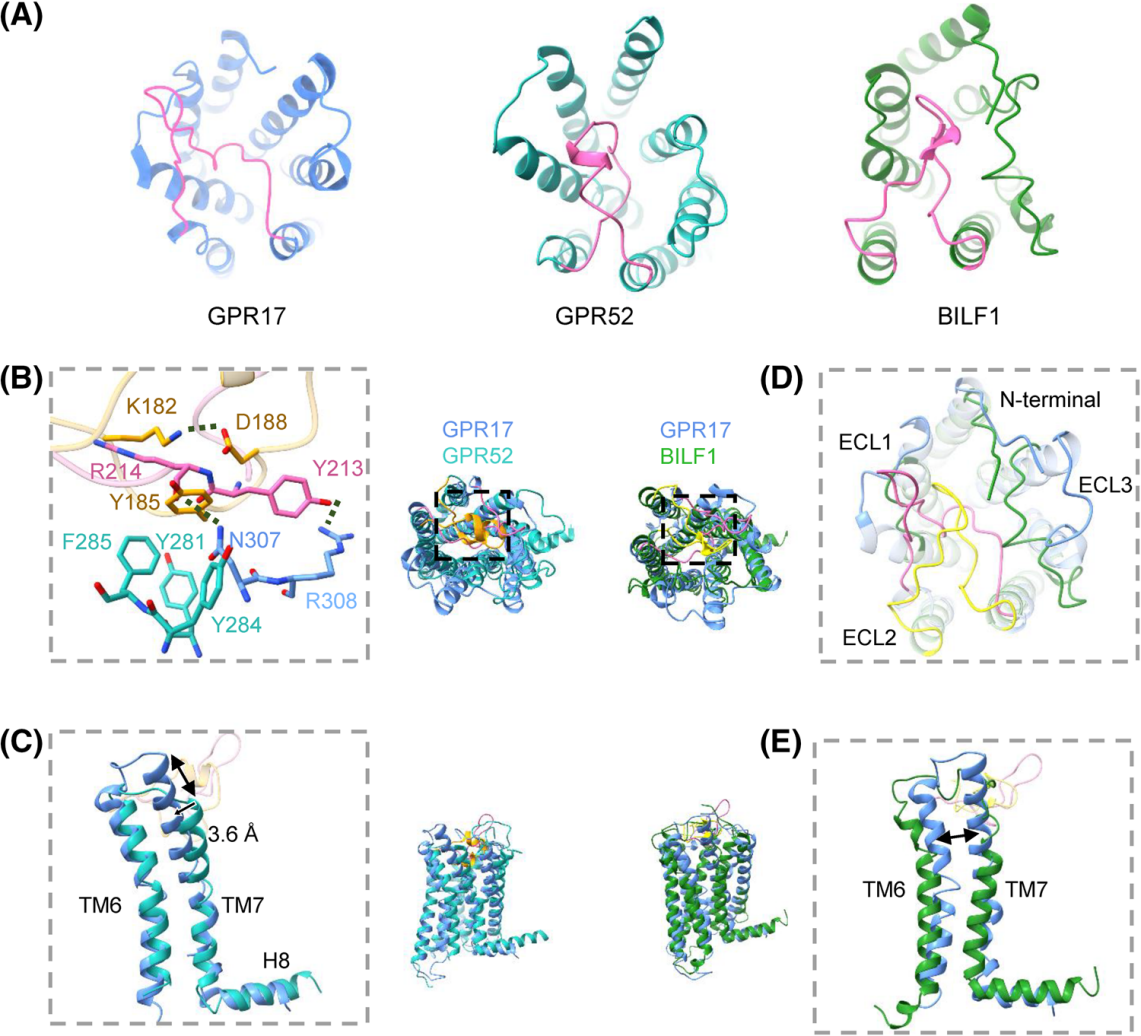
Cryo‐electron microscopy (EM) structure of GPR17 compared to self‐activated GPCRs. (A) Extracellular structures of GPR17 (cornflower blue), GPR52 (light see green; PDB: 6LI3), and BILF1 (forest green; PDB: 7JHJ). The ECL2s were highlighted in hot pink. (B and C) Superposition of GPR17 (cornflower blue) with GPR52 (light see green). The ECL2s of GPR17 and GPR52 were shown as hot pink and orange, respectively. Key residues were shown as sticks. Arrows indicated remarkable conformational changes. (D and E) Superposition of GPR17 (cornflower blue) with BILF1 (forest green). The ECL2s of GPR17 and BILF1 were shown as hot pink and yellow, respectively

In light of the previous studies suggesting the involvement of ECL2 in mediating self‐activation of GPCR, we compared the activated GPR17 structure with human GPR52 and Epstein‐Barr virus BILF1 (Figure 5A), which were reported self‐activating receptors.

On active GPR52, Y185^ECL2^ forms hydrophobic interaction with Y281^6.51^, Y284^6.54^, and F285^6.55^ of TM6. Moreover, Lys182^ECL2^ interacts with D188^ECL2^ via a salt bridge, which stabilizes ECL2 in the ligand pocket. Compared to GPR52, GPR17 has different interactions between ECL2 and TMs. There are hydrogen bonds between Y213^ECL2^ and R308^7.36^, R214^ECL2^ and N307^7.35^, which lead ECL2 to occupy the ligand binding pocket deeply (Figure 5B). The unique ECL2 conformation of GPR17 has not been observed in other GPCRs. However, the TM6, TM7, and H8 of GPR17 have similar conformation with GPR52 as well as other active GPCRs, which indicates that GPR17 is also at the fully active state (Figure 5C).

Self‐activated BILF1 structure was reported by K. Christopher Garcia group. BILF1 has two long ECLs: ECL2 (19 residues) and ECL3 (21 residues). ECL2 and ECL3 capping the entrance of BILF1 orthosteric pocket may hinder the assessment of diffusible ligands. However, the ECL2 of BILF1 is important to its protein expression but not significant for Gi signaling. Compared to BILF1, GPR17 has a longer ECL2 to deeply insert the orthosteric ligand binding pocket and be essential for coupling to Gi protein (Figure 5D). GPR17 has a narrower gap between TM6 and TM7, which suggests the gap of GPR17 is unlikely to be a similar ectopic binding pocket as BILF1 (Figure 5E). Compared with self‐activated GPCRs that have been previously described, these results confirm that the interactions of ECL2 with TM7 are important for GPR17 activation and structural stabilization.

### 2.5 Comparing the Gi‐binding interface of GPR17 with other Gi‐coupled GPCR

**FIGURE 6:**
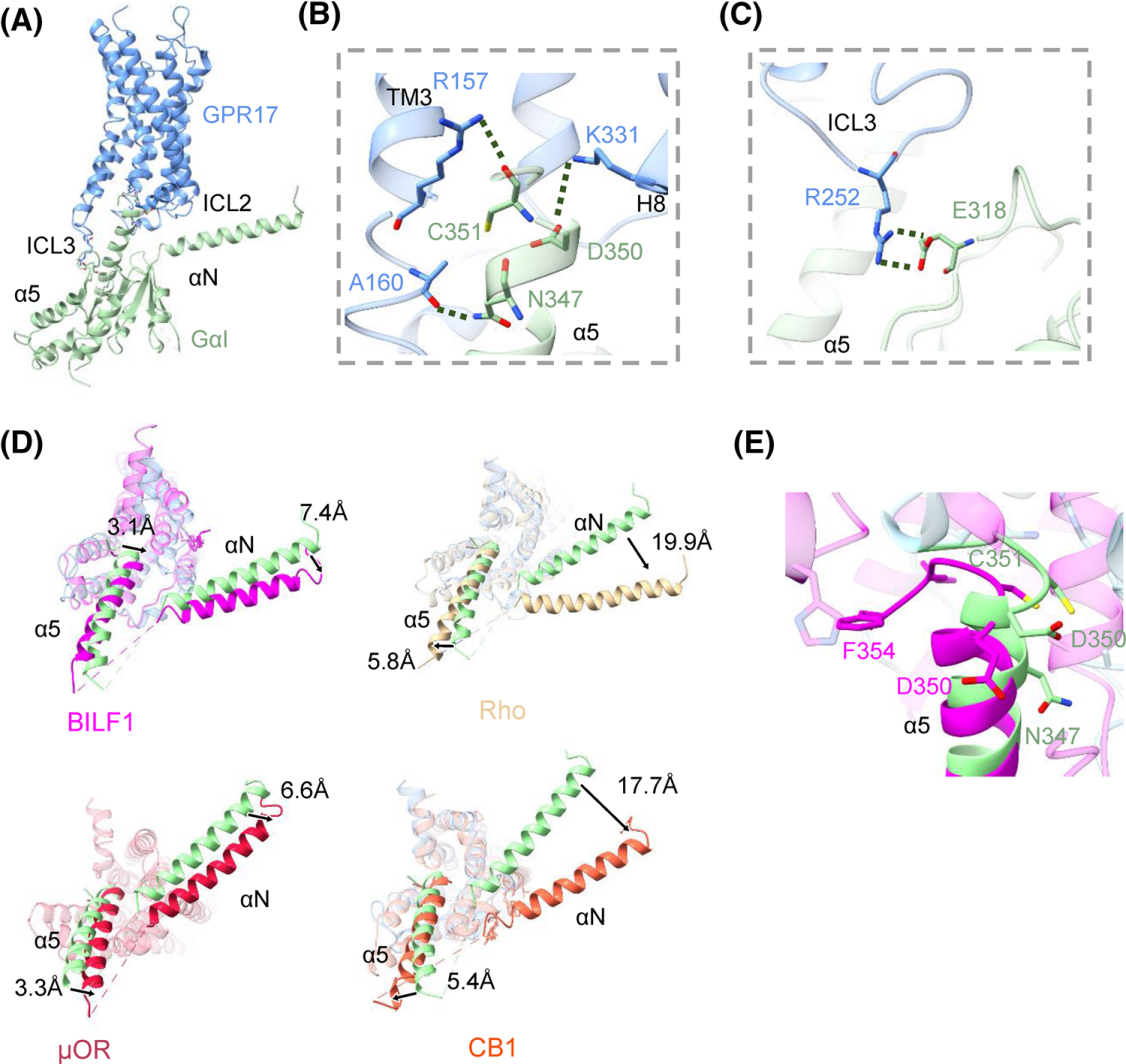
Analysis of the GPR17‐Gi interface and comparison with class A GPCRs. (A) Side view of the GPR17‐Gαi complex (cornflower blue‐pale green). (B and C) Key molecular interactions between in the GPR17 and Gαi. The residues of GPR17 and Gαi are shown as cornflower blue and pale green sticks. The hydrogen bonds were dark green‐dashed lines. (D) Alignment of GPR17 (cornflower blue‐pale green) with BILF1 (magenta, PDB: 7JHJ), Rhodopsin (Roh; gold; PDB: 6CMO), μ‐opioid receptor (μOR; crimson; PDB: 6DDE), cannabinoid receptor‐1 (CB1; tomato; PDB: 6N4B) complexed with Gαi. Their structural superimposition was displayed at a top‐down view. Arrows indicated remarkable conformational changes. (E) Structural comparison of the GPR17‐Gαi complex (cornflower blue‐pale green) and BILF1‐Gαi complex (magenta). The key residues of GPR17‐Gαi and BILF1‐Gαi complex were shown as sticks in pale green and magenta

GPR17‐Gi adopts nearly the same conformation compared to the recently reported Gi‐coupled class A GPCRs. The carboxyl‐terminus of Gi is buried in the helical core of the GPR17 transmembrane region formed by hydrogen bonds. The Gi‐coupled interface on class A GPCR (BILF1, Rho, μOR, CB1) is composed of TMs, ICL2 and ICL3.^15^, ^17^, ^18^, ^19^ For GPR17, ICL2 does not have interaction with αN of Gαi, but ICL3 indeed interact with α5 helix (α5) on Gαi (Figure 6A). The main interaction sites between GPR17 and Gαi are composed of hydrogen bonds and salt bridges in the interface of GPR17 and α5 of Gαi. C351 of Gαi forms a hydrogen bond with R157^3.50^ on TM3 of GPR17. Another hydrogen bond is found between N347 of Gαi and A160^3.53^ of GPR17 in close proximity to the E/DRY domain. Moreover, D350 on Gαi can form a salt bridge with K331^8.49^ on GPR17 Helix8 (Figure 6B). Interestingly, there is also a salt bridge between E318 on Gαi and R252 of ICL3 on GPR17, indicating a critical binding site of GPR17 to Gi (Figure 6C). We compared the structure of the GPR17‐Gi complex with those Gi‐coupled BILF1, Rho, μOR, and CB1 based on the alignment of receptors. When receptors were aligned, the relative orientations of α5 were similar, but the deflections of αN were large. Relative to the αN of Gi in the GPR17‐Gi, the αN of Gαi rotates ∼7.4 Å in the BILF1‐Gi, ∼19.9 Å° in the Rho‐Gi, ∼6.6 Å° in the μOR‐Gi, and ∼17.7 Å° in the CB1‐Gi measured at the residue A11 of Gαi (Figure 6D). If only aligns the Gi, the most regions of Gi were aligned well, and the α5 and αN showed similar conformations (Figure S3). But, we observed that D350 of Gαi has large deflection compared to BIFL‐Gi complex, suggesting that D350 of GPR17‐Gi plays a key role in the Gi‐binding interface (Figure 6E). Together, these results show that the interaction between GPR17 (ICL3, TM3 and H8) and Gαi is considered to stabilize the GPR17‐Gi complex.

## 3 DISCUSSION

So far, BILF1 and GPR52 are reported to be activated by their own structural elements.^15^, ^20^ GPR52 is an orphan GPCR in class A with high constitutive activity under physiological conditions. Similar to GPR52, constitutive BILF1 activity is noted, and cognate ligands are yet to be discovered. In GPR17, ECL2 functions as a self‐agonist which activates the Gi signaling. This is in contrast to the BILF1, whose ECL2 has limited effects on its signaling but is essential for the protein expression.

In GPR52, ECL2 functions as a self‐agonist, which activates the orphan GPR52. Our observation suggests that ECL2 of GPR17 occupies the orthosteric pocket to induce high level of basal signaling, which confirms a similar activation mechanism to GPR52. However, different to GPR52, the residues of GPR17 ECL2 interact with TM3 and TM7 residues via hydrogen bonds but not hydrophobic interaction and salt bridge.

What are the implications for ligand identification and drug development based on the structure of GPR17? The orthosteric pocket is occupied by ECL2 (CLQLYRE) and forms various interactions with hydrophilic residues R115^2.60^, Y118^2.63^, C132^3.25^, T135^3.28^, G136^3.29^, Y140^3.33^, Y279^6.51^, R283^6.55^, Y286^6.58^, R291^6.63^, N307^7.35^, and R308^7.36^. These results indicate a hydrophilic environment of the orthosteric binding pocket and thus the endogenous ligands of GPR17 might be hydrophilic molecules.Therefore, as GPR17 is expressed in neurons and oligodendrocyte progenitors, we suggest that future deorphanization work should focus on screening hydrophilic molecules in CNS. Also, the cryo‐EM structure of GPR17 we reported reveals the potential ligand‐binding pocket, which may benefit the further research on the leading compound screening to overcome the great challenge of neurodegenerative diseases.

GPR17 carries several microswitches, which are similar to the typical microswitches in other class A GPCRs. The DPxxY motif plays an essential part in GPR17 activation. The cyclic side chain of P^7.50^ maintains proper active conformation of TM7. DRF motif at the cytoplasmic end of TM3 is also present in CXC‐chemokine receptor 6 (CXCR6). Koenen et al. mutated CXCR6 DRF motif to the typical DRY and found no effect on ligand binding and receptor recycling.  Instead, DRF motif in CXCR6 may function as an environmental regulator, which alters calcium signaling capacity, migratory potential and chemotactic response compared to DRY containing CXCR6.^21^ Similarly, DRF‐containing GPR17 activation in oligodendrocyte precursors can stimulate cell migration, which functions as a post‐traumatic response.^22^ The highly conserved tryptophan (W^6.48^) in the CWxP toggle switch is substituted by F^6.48^ in GPR17, forming the CFxP motif. W^6.48^ controls TM6 movement, essential for ligand‐induced receptor activation and ionic lock inactivation. In the A3 receptor, W243F mutation alters downstream signaling via G protein or β‐arrestin.^23^, ^24^ In GPR17, the rotamer toggle switch at F^6.48^ forms a noncovalent contact interface with TM7 shaping the development of active conformation. The unique motifs on GPR17 may account for the auto‐activation features of GPR17 and warrant further investigation.

In a word, our results have profound implications for understanding the structural basis underlying mechanism of GPR17 and may provide rational, structure‐based, approaches for ligand screening on GPR17 for the treatment of neurodegenerative diseases. Moreover, the GPR17 structure in ligand‐free state complements the self‐activation mechanism in the orphan GPCR field. In light of the therapeutic essential of GPR17, revealing the structural change from inactive to active state is under process. Elucidation of the structures of GPR17 and its endogenous ligands may need further research.

## 4 MATERIAS AND METHODS

### 4.1 Cloning and purification of GPR17‐Gi complex

The full‐length human GPR17 was cloned into pFastbac A vector (Thermo Fisher Cat# 10360014) with N‐terminal hemagglutinin (HA) signal sequence, FLAG epitope, and 3C cleavage site, as well as a C‐terminal His tag. The construction of the dominant‐negative Gαi1 (DNGαi1) with mutations (G203A and A326S) was identical to that of GPR17. Whereas the Gβ1γ2 was cloned into the pFastbac Dual vector. The Bac‐to‐Bac baculovirus expression system (Thermo Fisher Cat# 10360014) was used to co‐express GPR17, DNGαi1, and Gβ1γ2 in *Spodoptera frugiperda* Sf9 cells (Invitrogen Cat# A35243), which were grown in suspension at 27°C. Sf9 cells at 4 × 10^6^ cells/ml density were infected with baculoviruses expressing GPR17, DNGαi1, and Gβ1γ2 at a ratio of 10:10:1. After 48 h, Sf9 cells were collected by centrifugation at 4000 g for 10 min and then stored at −80°C before protein purification.

Cell pellets were thawed and suspended by spinning in lysis buffer (10 mM Tris pH7.5, 0.5 mM EDTA) at 4°C for 60 min to facilitate complexes formation. Cell membranes were collected by centrifugation, and the supernatant was discarded. The sediment was homogenized by the dounce homogenizer in solubilization buffer containing NH buffer (20 mM HEPES pH 7.5, 100 mM NaCl), 10% glycerol, 10 mM MgCl_2_, 5 mM CaCl_2_, 1 mM MnCl_2_, 100 μg/ml benzamidine (Sigma–Aldrich Cat# 12072), 0.2 μg/ml leupeptin (Sigma‐Aldrich Cat# L5793), 25 μU/ml apyrase (NEB Cat# M0398S), and 100 μU/ml lambda protein phosphatase (NEB Cat# P0753S). Then, 1% DDM (n‐Dodecyl‐β‐D‐Maltoside, Anatrace Cat# D310) and 0.1% CHS (Sigma Cat#C6512) were added to the homogenate liquid and solubilized at 4°C for 2 h.

After centrifugation, the supernatant was collected, added with 2 mM CaCl_2_ and loaded onto the anti‐Flag M1 antibody affinity resin. The M1 resin was extensively washed with NH buffer containing 0.1% DDM, 0.01% CHS, and 2 mM CaCl_2_. After exchanging the 1% DDM buffer to 0.1% LMNG (Cat# 4216588) buffer with gradient steps, 10 × CMC buffer (NH buffer, 0.01% LMNG, 0.001% CHS, and 2 mM CaCl_2_) was used to further wash the M1 resin.

The GPR17‐Gi protein was eluted using 10 × CMC buffer without CaCl_2_ supplemented with 200 μg/ml Flag peptide (Sigma Cat# F3290), 5mM EDTA and 0.033% GDN (Anatrace Cat#GDN101).

The eluted protein was concentrated with Ultrafiltration tube (Merck, Amicon Ultra‐15ML) and incubated with the antibody fragment scFv16 for 2 h on ice at the molar ratio of 1:1.5.^18^ The GPR17 complex was further purified with Superdex 200 Increase 10/300 column (GE Healthcare) that was pre‐equilibrated with 10 × CMC buffer with 0.033% GDN. The pure GPR17 complex was concentrated with an ultrafiltration tube and flash frozen in liquid nitrogen for further use.

### 4.2 Negative staining EM

The carbon‐coated copper grid (zhong jing ke yi) was predischarged by glow discharge Easyglow and incubated with the GPR17 complex (0.05 mg/ml) for 2 min. Excess protein was removed using a filter paper gently. Immediately after blotting, the grids were stained with uranyl acetate (0.1 mg/ml) for 1 min, and the excess liquid was removed with filter paper before air‐dried. Data were collected using Talos 120C at 120 kV.

### 4.3 Cryo‐grid preparation and EM data collection

The Au 300 girds (QUANTIFOIL R 1.2/1.3) were discharged with Tergeo plasma cleaner. A droplet (3 μl) of purified 5.6 mg/ml GPR17‐Gi‐scFV16 complex was applied to the grid. Wait and blot for 3.5 s respectively at 10°C and 100% humidity using the Freezing plunger Vitrobot I (Thermo Fisher Scientific). Grids were quickly frozen in liquid ethane and transferred to liquid nitrogen for preservation until checked.

The grids were checked and cryo‐EM data were collected using the 300 kV Titan Krios Gi3 microscope (Thermo Fisher Scientific FEI). All movies were acquired at a magnification of 105,000 using the Gatan K3 BioQuantum camera. The raw pixel size corresponded to 0.85 Å. A GIF‐quantum energy filter (Gatan, USA) was used to remove inelastically scattered electrons. Movie stacks were automatically acquired with a 20 eV slit width and a defocus range from −1.0 to −2.0 μm. The exposure was 2.5 s, with frames collected every 0.05 s for total 50 frames per sample. The dose rate was 21.2 e/pixel/s. Automated single‐particle data acquisition was performed using SerialEM 3.7.

### 4.4 Image processing and 3D reconstruction

In total, 9513 movie stacks were aligned with dose‐weighted motion correction that was performed by cryoSPARC v3.3. The template was generated by 1007 manually picked particles using 81 projected images as reference after 2D classification. In the first‐round template picking, 8,276,241 particles were extracted. Then, the particles were processed to three cycles of 2D classification and reduced to 3,006,195. The particle size further was reduced to 730,103 after 3D classification. After the ab initio 3D constructions, 3D classes with 314,674 particles were subjected to homogeneous refinement, nonuniform refinement and local refinement. Finally, the density map with global resolution of 3.02 Å at FSC 0.143 was obtained.

### 4.5 Model building and refinement

The predicted human‐GPR17 structure obtained from Alphafold (https://alphafold.ebi.ac.uk/) was used as a template to build the GPR17‐Gi‐scFv16 complex model. The template for Gi‐scFV16 was obtained from the Gi heterotrimer of FPR2‐Gi cryoEM structure (PDB 6OMM). Model docking was carried out using Chimera.^25^ Manually adjustment and rebuilding was performed with COOT.^26^ The model was repeatedly refined using phenix.realspace refinement module in Phenix.^27^ The softwares, such as UCSF Chimera, UCSF ChimeraX, and PyMOL, were used to prepare the molecular graphics figures. The final model statistical analysis was performed by Phenix.

### 4.6 cAMP assay

First, the full‐length GPR17 with indicated point mutants were cloned into pcDNA3.1 vector with an N‐terminal Flag tag, and a 3C protease site inserted after a HA signal peptide. cAMP level with GPR17 plasmid DNA concentrations of 100, 200, 300, and 400 ng/well was analyzed using the cAMP‐Gi KIT (Perkin Elmer, TRF0263) according to the manufacturer's instructions. HEK‐293 cells (ATCC CRL‐1573) were seeded in 24‐well culture plates (at a density of 70%–90% cells per well). The cells were transiently transfected with WT or mutated GPR17 plasmids using Lipo3000 reagents (Invitrogen, L3000). After 36 h, the cells were washed with PBS twice and suspended with stimulation buffer containing 500 μM IBMX. The cell suspension was transferred to a 384‐well plate for 30 min at 37°C. Afterward, 12 μM forskolin was added to a 384‐well plate and incubated at 37°C with 5% CO_2_ for another 45 min. Then, at room temperature, 5 μl cAMP Eu‐cryptate reagent and anti‐cAMP‐d2 working solution were added to the 384‐well plate for and incubated for 1 h. The fluorescence signals were detected on the multimode plate reader (Perkin Elmer) at 620/665 nm. The experiments were performed in triplicate.

### 4.7 Cell surface expression assay

Expression levels of GPR17 plasmid in HEK‐293 were confirmed by flow cytometry. Briefly, transfected HEK‐293 cells were blocked with 5% BSA at room temperature for 15 min and labeled with anti‐FLAG antibody (1:100, Thremofisher) at 4°C for 1 h. The antibody‐stained cells were washed with PBS three times. After, the cells were incubated with anti‐mouse Alexa‐488‐conjugated secondary antibody (1:300, Beyotime) at 4°C in dark for 1 h. Approximately 10,000 cellular events were counted for each sample, and the fluorescent intensity was quantified by BD Accuri C6 Plus flow cytometer. The experiments were repeated at least three times.

### 4.8 Statistical analysis

Data were presented as mean ± SEM (standard error of the mean) in triplicates, and the statistical significance was shown in figure legends. Statistical analyses were performed with GraphPad Prism 9.0 with One‐way analysis of variance followed by Tukey's multiple comparisons test. Values with *p* < 0.05 were considered statistically significant, and ns means not significant.

## CONFLICT OF INTEREST

The authors declare no conflict of interests.

## AUTHOR CONTRIBUTIONS

F. Y., Z. Z., B. Z., S. G., W. G., Z. W, and J. L. performed the experiments, collected, and analyzed the data. Y. D. and G. C. decided his research direction and supervised the project. F. Y., T. W., and G. C. wrote the manuscript. X. P., and B. Z. revised the manuscript. All authors discussed the results and reviewed the manuscript.

## Supporting information

Supporting Information

## Data Availability

The data supporting this study are available from the corresponding author upon reasonable request. The cryo‐EM density map and structure coordinate of GPR17‐Gi complex have been deposited to the Protein Data Bank database (PDB) and Electron Microscopy Database (EMDB) under accession codes 7Y89 and EMD‐33682, respectively.
