# Supplementary material for: Cryo‐EM structure of G‐protein‐coupled receptor GPR17 in complex with inhibitory G protein
Source: MedComm (2020). 2022 Sep 10;3(4):e159. doi: 10.1002/mco2.159 (PMC9464062; doi:10.1002/mco2.159)
Supplement: Supplementary file 1 — Supporting Information [file MCO2-3-e159-s001.docx]

**Supplementary materials**

**Cryo-EM Structure of G-protein-coupled Receptor GPR17 in complex with inhibitory G protein**

Fang Ye^1,2,#^, Thian-Sze Wong^1,#^, Geng Chen^1,#^, Zhiyi Zhang^1^, Binghao Zhang^1^,Shiyi Gan^1^, Wei Gao^1^, Jiancheng Li^3^, Zhangsong Wu^1^, Xin Pan^1^, Yang Du^1,2*^

^1^ Kobilka Institute of Innovative Drug Discovery, Shenzhen Key Laboratory of Steroid Drug Discovery and Development, School of Medicine, the Chinese University of Hong Kong, Shenzhen 518172, Guangdong, China

^2^ The Chinese University of Hong Kong, Shenzhen Futian Biomedical Innovation R&D Center, Shenzhen 518060, Guangdong, China

^3^ Instrumental Analysis Center, Shenzhen University, Shenzhen 518060, Guangdong, China

***Correspondence**

Yang Du, Kobilka Institute of Innovative Drug Discovery, Shenzhen Key Laboratory of Steroid Drug Discovery and Development, School of Medicine, The Chinese University of Hong Kong, Shenzhen, Guangdong, 518172, China.

Email: [yangdu@cuhk.edu.cn](mailto:yangdu@cuhk.edu.cn)

^#^ Fang Ye, Thian-Sze Wong, Geng Chen contributed equally to this paper.

**Supplementary Materials**

**Table S1. Statistics for Cryo-EM data collection and processing**

| **Protein** | **GPR17-G_i_-scFV16** |
| --- | --- |
| **Cryo-EM Data Collection** |  |
| Voltage (kV) | 300 |
| Magnification (x) | 105,000 |
| Pixel size (Å) | 0.85 |
| Electron exposure (e^-^/ Å^2^) | 52.5 |
| Defocus range (μm) | [-1.0, -2.0] |
| Number of image stacks | 9,513 |
| Number of frames per stack | 50 |
| **Cryo-EM Data Processing** |  |
| Initial number of particles | 8,276,241 |
| Final number of particles | 314,674 |
| Map sharpening B factor (Å^2^) | -50 |
| Map resolution (Å) | 3.02 |
| Map resolution range (Å) | 2.5-4.5 |
| FSC threshold | 0.143 |
| **Model Refinement** |  |
| Model resolution range  Number of amino acids | 3.5-9  1127 |
| Total non-hydrogen atoms | 8756 |
| Bond length r.m.s.d. (Å) | 0.004 |
| Bond angle r.m.s.d. (°) | 1.016 |
| Ranmachandran Plot |  |
| Favored (%) | 93.79 |
| Allowed (%) | 6.21 |
| Outliers (%) | 0.00 |
| Rotamer outliers (%) | 0.12 |
| MolProbity score | 1.98 |

**
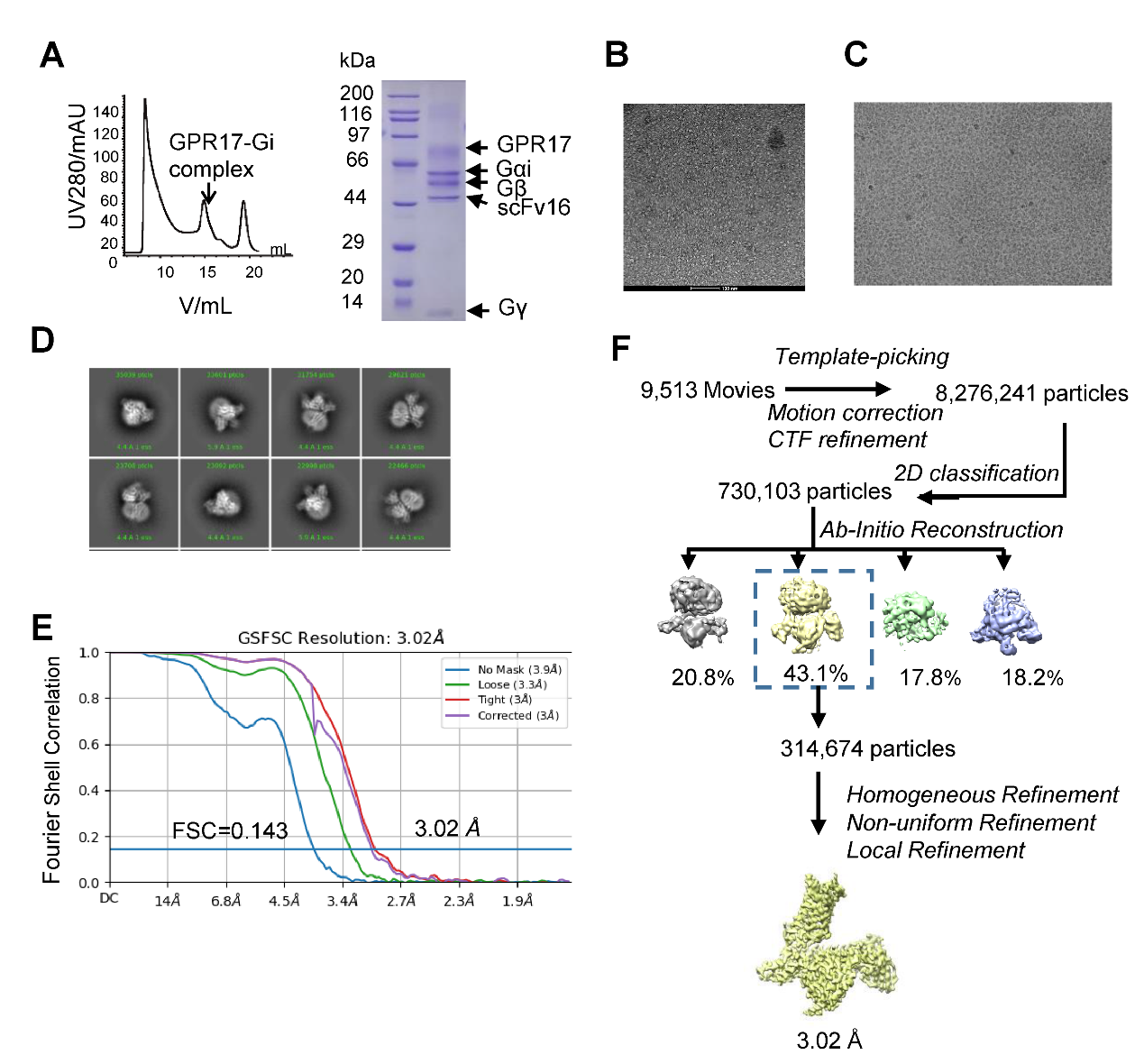
**

**FIGURE S1. Sample preparation and cryo-EM structure determination.** (A) Size exclusion chromatography profile of the GPR17-Gi complex. (B) the complex was assessed by the negative stain EM. (C) Representative micrograph of the complex particles. (D) Representative 2D classification result. (E) Fourier shell correlation (FSC) curve with the estimated resolution according to the gold standard with a mask excluding detergent micelles. (F) Cryo-EM image processing workflow for image processing. the Methods section provided the details.


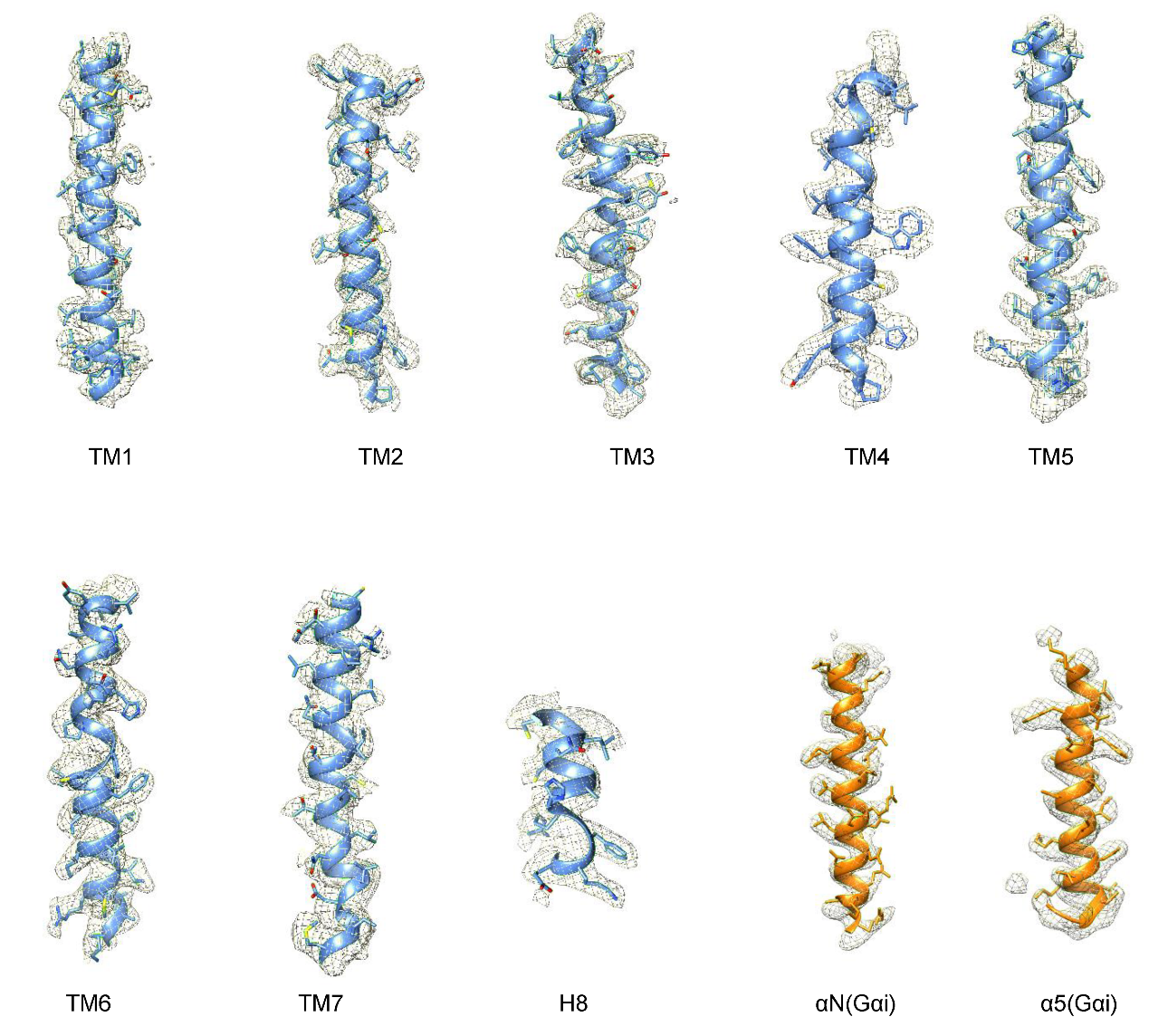


**FIGURE S2. Representative Cryo-EM map for key components of the GPR17-Gi complex.** Representative density maps and models for TM1-7 and H8 of GPR17, and the N-terminal and C-terminal α helice of Gαi (αN and α5).


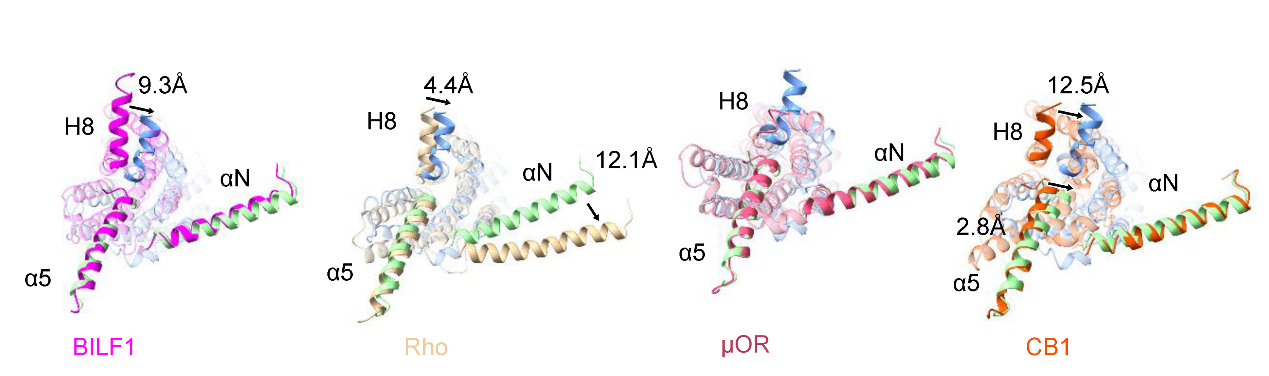


**FIGURE S3.** **Structural comparison of GPR17-Gi, BILF1-Gi, Rho-Gi, μOR-Gi and CB1-Gi.** Alignment of GPR17-Gαi complex (cornflower blue-pale green) with BILF1 (magenta, PDB: 7JHJ), Rhodopsin (Roh; PDB: 6CMO), μ-opioid receptor (μOR; crimson; PDB: 6DDE), cannabinoid receptor-1 (CB1; tomato; PDB: 6N4B) complexed with Gαi. Their structural superimposition was displayed at a top-down view. Arrows indicated remarkable conformational changes.
